# A study of forecasting tennis matches via the Glicko model

**DOI:** 10.1371/journal.pone.0266838

**Published:** 2022-04-08

**Authors:** Jack C. Yue, Elizabeth P. Chou, Ming-Hui Hsieh, Li-Chen Hsiao

**Affiliations:** 1 Department of Statistics, National Chengchi University, Taipei, Taiwan; 2 Physical Education Office, National Chengchi University, Taipei, Taiwan; LUMSA: Libera Universita Maria Santissima Assunta, ITALY

## Abstract

Tennis is a popular sport, and professional tennis matches are probably the most watched games globally. Many studies consider statistical or machine learning models to predict the results of professional tennis matches. In this study, we propose a statistical approach for predicting the match outcomes of Grand Slam tournaments, in addition to applying exploratory data analysis (EDA) to explore variables related to match results. The proposed approach introduces new variables via the Glicko rating model, a Bayesian method commonly used in professional chess. We use EDA tools to determine important variables and apply classification models (e.g., logistic regression, support vector machine, neural network and light gradient boosting machine) to evaluate the classification results through cross-validation. The empirical study is based on men’s and women’s single matches of Grand Slam tournaments (2000–2019). Our analysis results show that professional tennis ranking is the most important variable and that the accuracy of the proposed Glicko model is slightly higher than that of other models.

## Introduction

Data analytics has become very popular due to the rapid development of big data. Organizations and individuals create more opportunities by making use of the available information. For example, industries utilize big data to attain more customers and higher profits, while governments exploit big data to obtain more efficient resource allocations and better operations. Ever since IBM proposed that there are four characteristics of big data, namely, the 4 Vs (volume, velocity, variety, and veracity), many researchers have suggested expanding the characteristics of big data. Value, variability, and viability are some examples of new Vs. Nonetheless, the key to applying big data is to uncover the patterns hidden in the data, regardless of how many Vs are involved.

Ideally, we would apply data-driven techniques to discover the value of data and let the data tell the story. However, in practice, it is difficult to rely solely on automated data analytics, i.e., performing data analysis with little human intervention, since the process of selecting important variables depends on the study objective and domain knowledge. For example, suppose we are interested in professional tennis matches. If we want to predict the winners of matches, the related variables may include the players’ tournament history and personal information [1, 2]. On the other hand, if our focus is on the upset probability of matches, then we may want to consider other variables, such as the match records against common opponents and the format of matches [3, 4].

In reality, it is not possible to record all the available information, and it is also unlikely that it is all available digitally. Even though we can collect as many materials as possible, another question arises: should we include all variables in the study? In other words, the consideration is whether more data are better or if we can distinguish signals from noise. Answering this question is not easy, and it somewhat influences how we deal with the problem. In the case of predicting the outcomes of tennis matches, most studies plug all available variables into models (especially machine learning models) without exploring the characteristics of input variables for example, [5–7], although this in some way conflicts with how we conduct statistical analysis.

Selecting appropriate models is also a critical issue in predicting match results, in addition to recognizing important variables. The models for predicting tennis matches can be separated into three categories: regression-based, point-based, and paired comparison [8]. We can use macro- and microlevel forecasts [9] as an analogue to describe the difference between regression- and point-based models. It is not necessarily that microlevel models can provide more accurate predictions, which would depend on the study goal and data attributes. In tennis match prediction, regression-based models are the most popular, while point-based models tend to have less accurate predictions [10].

In order to predict the tennis match results, researchers have considered various predictors into their classification algorithms. Some of the relevant variables are player’s physical and mental characteristics, such as age, height, handedness, and career wins [11, 12]. The results of the games are also considered as predictors, like winning percentages on the first, second, and return serve, total point win, and different type of court [1]. In addition, tournament factors and some indicators from the matches are also used. Furthermore, the most common predictor for the models is player’s rank [8].

In this study, we aim to evaluate the prediction accuracy of regression-based models, given the same variables, and explore whether introducing new variables can improve model prediction. We generate new variables via a Bayesian approach, namely, the Glicko model, which was originally used in professional chess. In addition, we will apply tools of exploratory data analysis (EDA) to identify important variables. These variables will be plugged into statistical models and machine learning models to check which models produce more accurate results. The model comparison is based on the single matches of Grand Slam tournaments (2000–2019), and error calculation is via cross-validation. In the rest of this paper, we first introduce the data and models used in the next section, followed by the key findings of EDA in Section 3. We show the results of the model comparison in Section 4 and provide our suggestions in Section 5.

## Data and methodology

**Fig 1:**
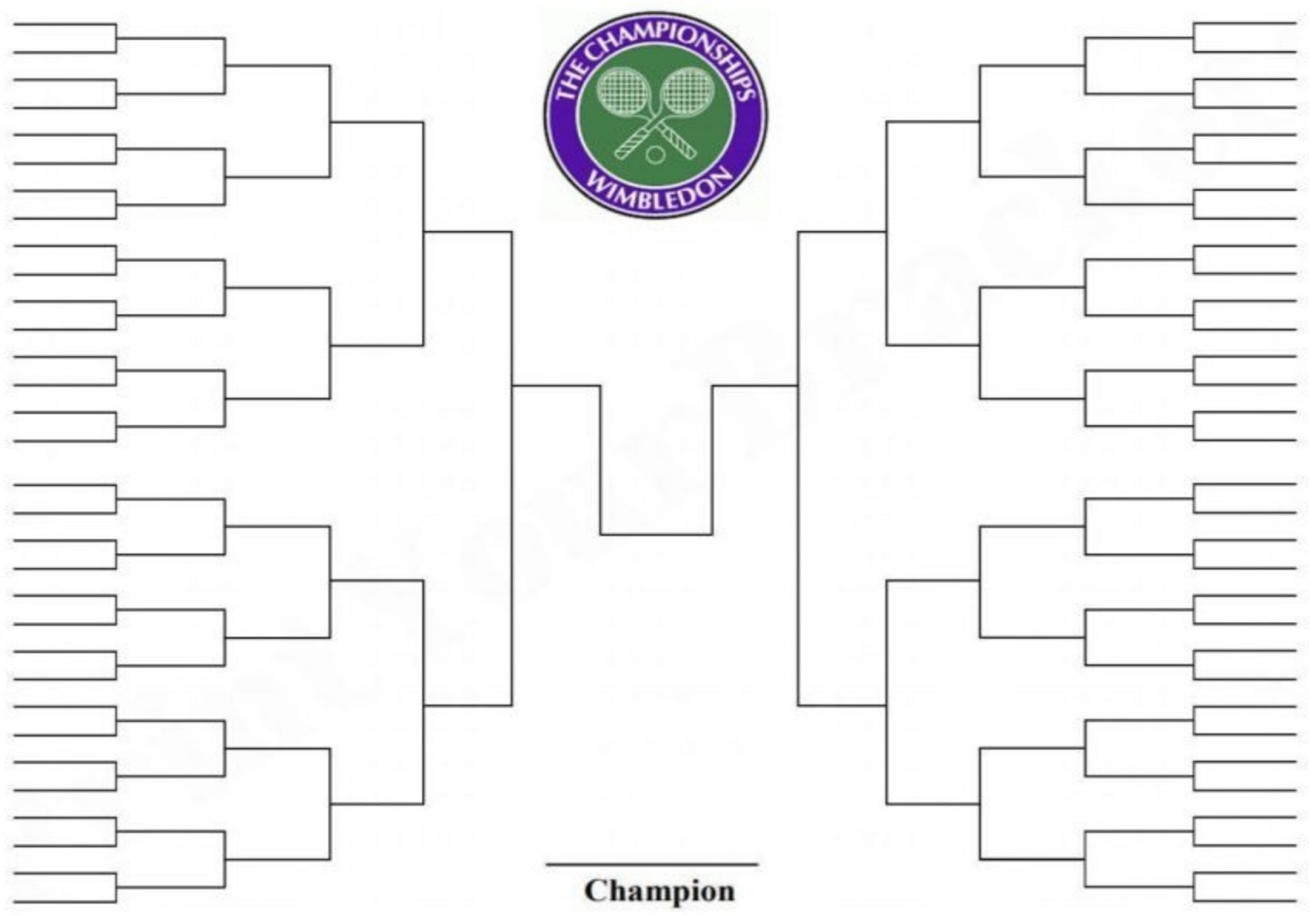
Wimbledon championship bracket. https://www.interbasket.net/wp-content/uploads/Wimbledon-bracket-1-of-2-print-768x590.jpg.

We study the single matches of Grand Slam tournaments, namely, the Australian Open, the French Open, Wimbledon, and the US Open, which are the world’s four most important annual tennis events. For each Grand Slam tournament, there are 7 rounds of single-elimination games (1st round, 2nd round, 3rd round, 4th round, quarter-final, semi-final, and final), with a total of 127 matches (and 128 players). Fig 1 shows the bracket of single matches in Wimbledon. Grand Slam tournaments seed the players according to their rankings and currently adopt the 32-seed format. We consider 20 years of Grand Slam single matches (2000–2019) for men and women in this study and use the data collected (49 variables) from GitHub, which are organized by Jeff Sackmann at https://github.com/JeffSackmann.

Judging from how Grand Slam tournaments seed the bracket, it seems that the ranking is believed to be a reliable index for measuring players’ strength. We will adapt this notion and introduce a rating index for predicting the results of tennis matches. The current ranking method used by the Association of Tennis Professionals (ATP) and the Women’s Tennis Association (WTA) is to determine the seeding of players and qualification for entry in a tournament. Players’ ranking points are decided by the stage of tournament they reached and the reputation of the tournament they participated in for the last 52 weeks. In other words, the ranking points will not be considered if players did not play for the last 52 weeks. The ranking points of tennis tournaments can be found on the official websites of the ATP and WTA.

Elo ratings [13] is a famous rating system for professional tennis and was initially used in rating chess players. This chess rating system has been used successfully for paired comparison in sports; it measures players’ strength based on their past performance in the sequence of matches, and ratings are updated after each competitive result. Many studies proved the efficiency and accuracy of Elo ratings in predicting the winner of tennis matches [8], and it is further extended for in-play tennis betting [14]. [15] used Elo-based ratings to predict ratings and proved that it works better than the official rating system. Additionally, the Elo rating system uses the normality assumption.

Another rating system is the Glicko model [16], developed by Mark Glickman to improve the Elo rating system. Three significant adjustments are involved in the Glicko formula, including an additional parameter presented as a standard deviation—rating deviation (*RD*), a time factor, and opponents’ ratings and deviations. *RD* represents variability/uncertainty in the rating. The deviation will increase at the beginning of the updating period and decrease at the end. Uncertainty should decrease and ratings become more stable as more games are played or the ratings converge to the player’s actual rating. The time factor in the Glicko system allows an increase in *RD* when a player has not played any rated game for a certain period, representing a higher uncertainty of the rating accuracy than a player playing regularly. The third factor takes opponents’ ratings and *RD*s into consideration.

The Glicko rating system is a paired comparison model for a player and his/her opponents in a rating period. A player’s rating, *θ*, is assumed to be a random variable that follows a normal distribution with the players’ average strength (*r*) and the degree of uncertainty in the players’ strength (*RD*) known, *θ*|*r*, *RD*^2^ ∼ *N*(*r*, *RD*^2^). The distribution represents the expected performance of a player. This paired comparison model allows *r* and *RD* to vary over time. The system adapts the Bayesian approach, modelling the game outcomes by the Bradley-Terry model [17]. The first step in the Glicko algorithm is to determine *RD*. Before entering a new rating period, the rating deviation will increase by a constant over time, *c*^2^ *t*.

$$RD=\text{min}(\sqrt{RD_{old}^{2}+c^{2}t},RD_{0})$$

where *RD*_0_ is the initial rating variance of a new player, *RD*_*old*_ is the current player’s original *RD*, *c* is a constant that represents how quickly the *RD* increases over time, and *t* is the number of rating periods since the last competition.

The Glicko rating system is an approximate estimation of a player’s true strength via a Bayesian approach. It can accumulate previous information and continuously update the parameters with new match results. The Bayesian estimation is generally straightforward in the case of a conjugate prior, i.e., prior and posterior distributions belonging to the same distribution. However, this is not the case for the Glicko model since the match results are binary. Because of the rapid development of computer technology, Bayesian estimation has become more convenient, and simulation-based MCMC (Markov chain Monte Carlo) methods can be used to obtain Bayesian estimates. In this study, the Glicko ratings were calculated using the “PlayerRatings” package [18] in R, with an initial mean strength (*r*) of 1500, a deviation (*RD*) of 350, and a constant (*c*) of 10, after various experiments.

## Exploratory data analysis

We will apply EDA tools to explore the tennis match data in this section. The goal of EDA is to detect important attributes of tennis matches and to identify useful variables for constructing predictive models. The chosen variables will be plugged into classification models, such as statistical and machine learning models, in the next section. The variables considered include players’ demographic information (e.g., residence, age, the year their career started, and the hand used) and the ranking data (rank and points) from 2000 to 2019. We aim to discover the relationships between player characteristics and match outcomes, which can provide explanatory power in relation to model effectiveness. Interestingly, we found that the Pareto principle (“80–20” rule) exists for the case where players’ advancement to later rounds is closely associated with their rankings. Pareto principle means that 80% of effects (winning results) come from 20% of causes (higher-ranked players). This can be adequately described by the power law.

**Fig 2:**
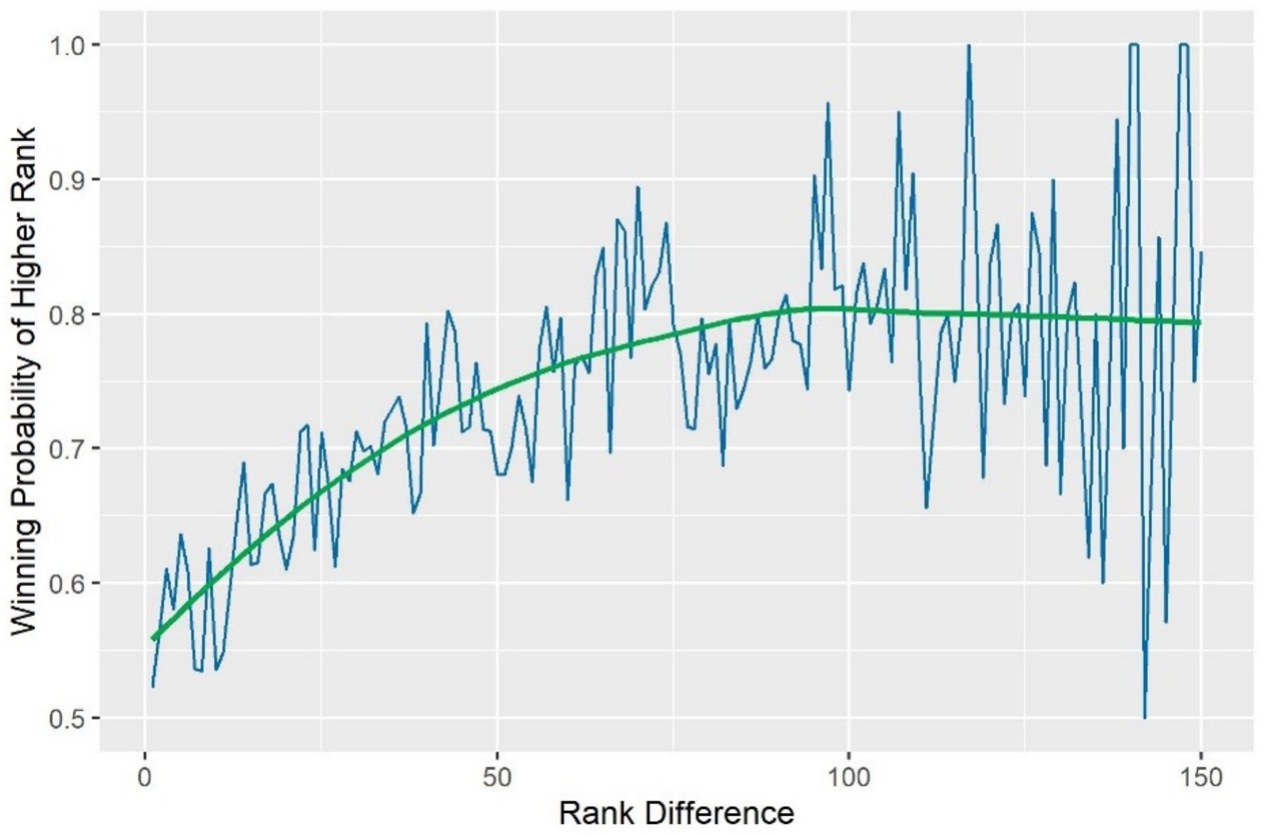
Winning probability of higher-ranked player vs. rank difference (male single).

**Fig 3:**
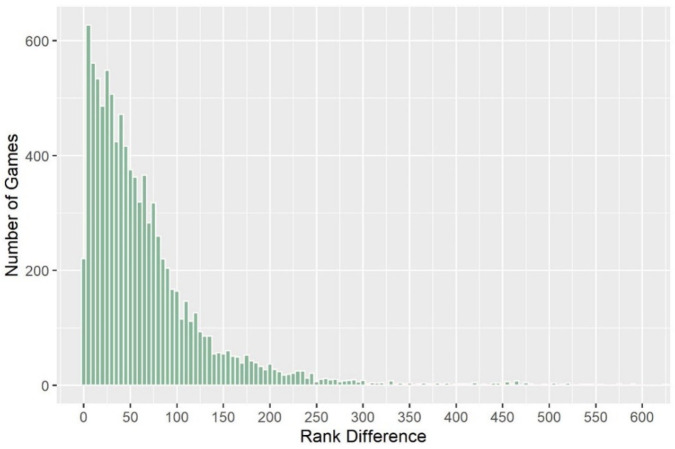
Histogram of ranking differences (ATP male).

We should first depict the relationship between the ranking and Grand Slam match result for male singles, since the ranking is believed to be a crucial factor and lower-ranked players defeating higher-ranked players are often called “upsets” (or shocks). Fig 2 shows the line chart of the ranking difference between higher- and lower-ranked players and the winning probability of higher-ranked players. Additionally, we apply the locally weighted regression (LOESS) method to smooth the winning probability. It appears that the winning probability increases with the ranking difference and levels off (to approximately 0.8) when the ranking difference reaches 80. Note that the results in Fig 2 imply that the predicted accuracy is very different for lower- and top-ranked players, similar to that in [8]. Additionally, the winning probability has higher fluctuations for larger ranking differences due to smaller exposures (or data points), which are shown in Fig 3.

The increasing trend of winning probability in Fig reffig:fig2 implies two possible further developments. First, the ranking is a fine indicator and can serve as a threshold for evaluating the prediction models of tennis matches, although the prediction accuracy is at 80% if relying solely on the ranking. We may want to include more variables to increase the prediction accuracy. The other consideration is that the probability of advancing to further rounds should also be related to the ranking. In other words, the players’ ranking is a critical factor for deciding whether they can reach later rounds. For example, we would expect that the probability of advancing to the semi-final is higher for the top 10 players than for the non-top 10 players. We will use the cumulative probability to demonstrate this phenomenon.

**Fig 4:**
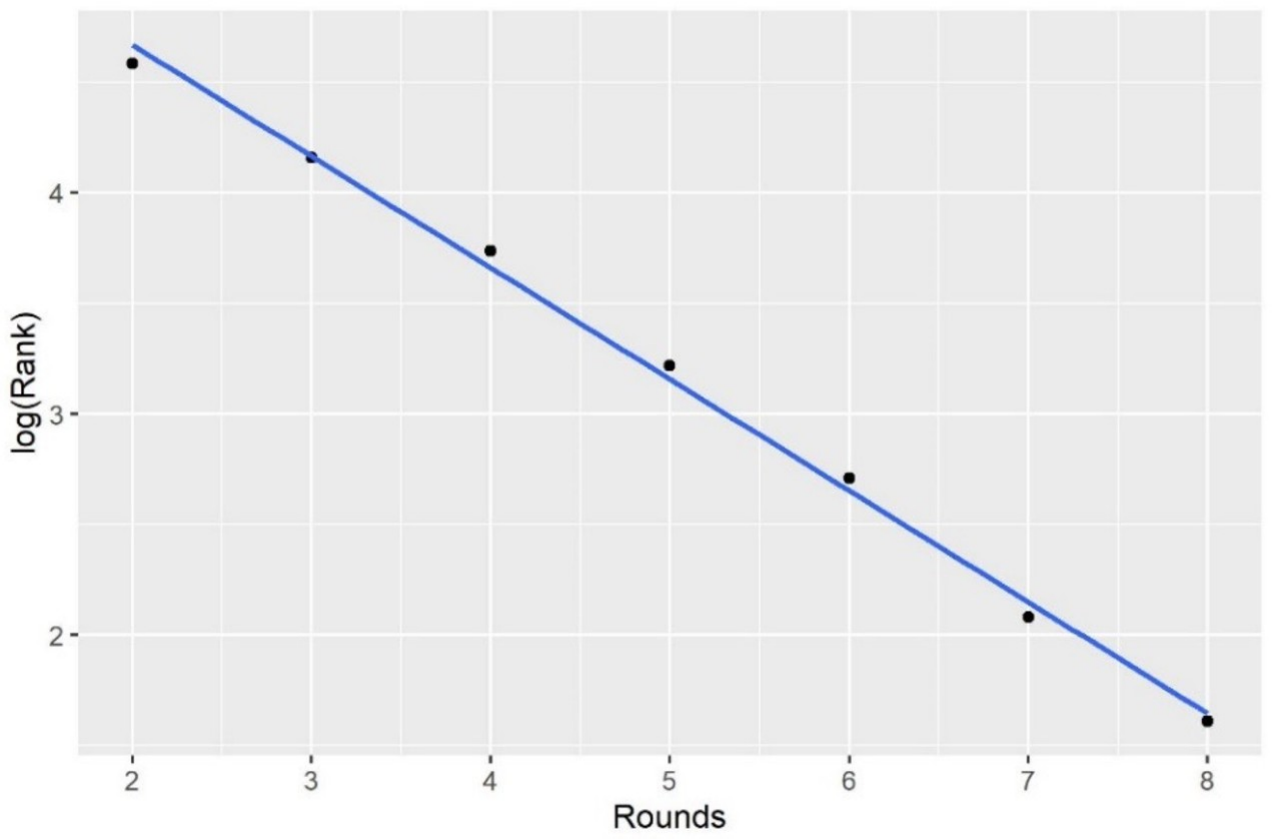
Logarithm of rank vs. rounds (80%).

$F_{X_{s}}\left(x\right)$ is the cumulative density function of *X*_*s*_, $F_{X_{s}}\left(x\right)=P\left(X_{s}\leq x\right)$, and *P*_0_ is the chosen percentile. Let the inverse distribution function be defined as

$$F_{X_{s}}^{-1}\left(P_{0}\right)=\text{inf}\left(x|F_{X_{s}}\left(x\right)\geq P_{0}\right)=x_{s}$$

where *x*_*s*_ is the lowest rank reaching round *s*, *s* = 1, 2, …, 7, and 7+, with 5, 6, 7, and 7+ indicating quarter-final, semi-final, final, and championship, respectively. In this study, we select *P*_0_ = 0.8, and the calculated results are shown in Table 1. For example, of the players who won the tournament, 80% were top 5 players. Similarly, 80% of finalists were top 9 players. The power law distribution can be used to describe the relationship between the lowest rank *x*_*s*_ and round *s*; i.e., the logarithm of *x*_*s*_ is a linear function of *s* (Fig 4).

**Table 1:** 80-percentile rank for each round (male single, 2000–2019).

Match	80-percentile *x*_*s*_	log(*x*_*s*_)	number of Samples
Champion	5	1.6094	80
Final	9	2.1972	160
Semi-Final	15	2.7081	320
Quarter-Final	25	3.2189	640
4th Round	41	3.7136	1280
3rd Round	64	4.1589	2560
2nd Round	98	4.5850	5120

The power law has been applied in many areas, such as physics, biology, and social science. For example, the Gutenberg-Richter law is a well-known power law, which is log_10_ *N* = *α* − *βM*, where *N* is the number of earthquakes larger than magnitude *M* and *α* and *β* are parameters [19]. This power law function fits well in our case, with an *R*^2^ larger than 0.9 if applying least square linear regression to derive the estimates of *α* and *β*. The power law indicates that the top players dominate the winners (finalists and semi-finalists) of Gram Slam tournaments, and the ranking would be an important variable if our goal were to predict the champion of a tournament. Note that we also tried other *P*_0_ values, but the fitting results were not as good as that of *P*_0_ = 0.8. This coincides with the concept behind the “80–20 Rule” (or Pareto principle).

**Fig 5:**
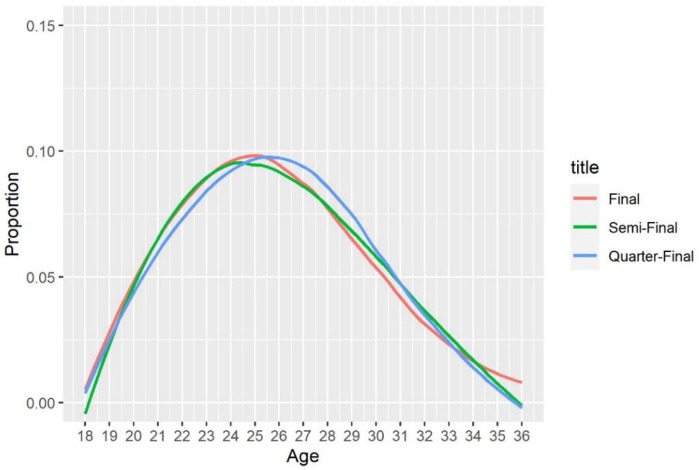
Age structure of players advancing to different rounds (male single).

We also explore other players’ variables (e.g., age, height, and professional years) and tournament information (e.g., hard, clay, and grass courts). Some of the variables have a strong connection with the winning probability, but the relationships between these variables and winning probability are not as strong. For example, in Fig 5, the age structures of players advancing to the final, semi-final, and quarter-final are very similar, with the peak at an age of 25 years for all 3 cases. Additionally, the winning probability of players is related to the type of courts. In any case, we will consider all available variables for statistical and machine learning models to predict the winning probability of Grand Slam matches in the next section.

**Fig 6:**
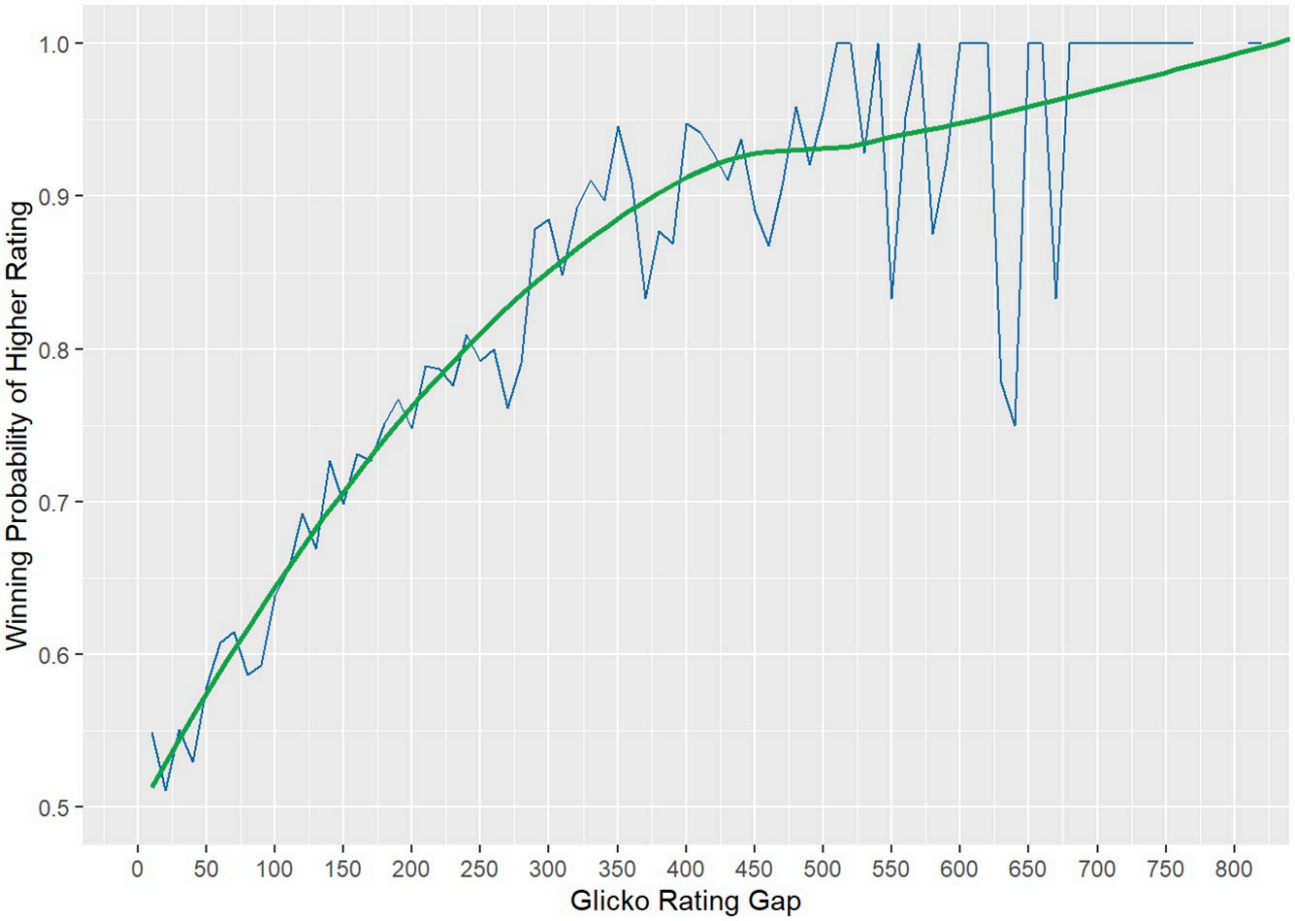
Winning probability of higher-rated player vs. rating difference.

In Fig 6, the bigger the Glicko rating differences between two players, the higher the winning probability of higher ranking players. The higher the winning probability of higher ranking players approaches 100% when the rating difference approaches 800. It confirms the proposed variable “Glicko rating” is essential.

After multiple experiments with cross-validation, the subset of the variables that produce the highest accuracy rate is recorded as our final model. For example, first, we applied all the variables in the ATP dataset to the logistic regression, and we found that these variables: “ATP points”, “ATP ranking”, “age”, and “professional years” are highly significant at a significance level of 0.01. The variables and hyperparameters we used in the models are summarized in S1 Table.

## Model comparison

We compare the performance of prediction models, including statistical and machine learning models, to the proposed model in this section. The empirical data considered are the single men’s and women’s matches of Grand Slam tournaments (2000–2019). These data are separated into training data and testing data, and the model performance is based on the prediction results of the testing data. We should use the ranking as a baseline model that predicts the winner as the player with the highest ranking. The statistical and machine learning methods considered are logistic regression, support vector machine (SVM), neural network, and LightGBM (light gradient boosting machine), which are often used to predict the winners of tennis matches. Basically, we apply all available variables to all models and use the training data to construct models. For prediction performance, we chose accuracy and area under the curve (AUC) as the evaluation criteria.

**Fig 7:**
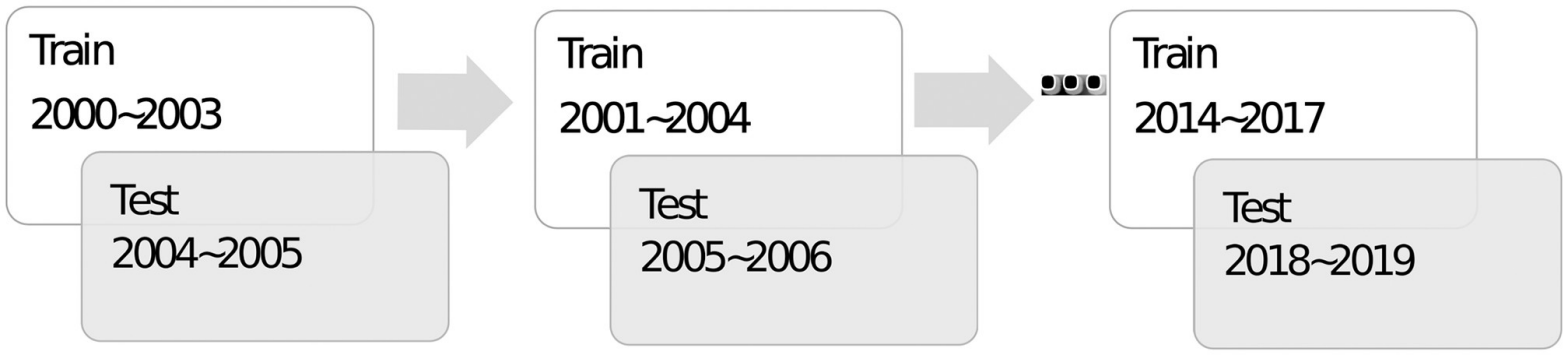
Possible combinations of moving windows.

For time sequence data, such as the 2000–2019 tennis matches considered in this study, there are two possible settings for applying cross-validation: moving window and non-moving window. The moving window technique is commonly used for time series data, and we choose the format of 4-year training data and 2-year testing data. For example, the first moving window is the 2000–2005 data, i.e., 2000–2003 as the training set and 2004–2005 as the testing set, and other moving windows follow the same rule. There are 15 possible combinations of moving windows, as illustrated in Fig 7. For the non-moving window, if applying the same 4/2 year setting of training/testing sets, we randomly select 4 years (out of 20 years) of data for the training set and 2 years out of the remaining 16 years as the testing set. Although we think that the prediction results of the moving window approach are more appropriate for evaluation, we will provide those of the non-moving window approach as a reference.

The proposed Glicko model considers the estimates of parameters in the Glicko model, in addition to other variables, in logistic regression. Note that the extra variables introduced by the Glicko model can be further expanded. For example, we found that the parameter estimates of the Glicko model are very different for the matches on clay courts compared to those on grass/hard courts. Thus, we can add court-related Glicko variables to the proposed model. The prediction results of men’s single matches (moving window) are shown in Table 2 (averages of 15 possible combinations). The differences in prediction accuracy between models are not very obvious, and even the baseline model (using the players’ rankings as the criterion) can reach 72.4% accuracy. Nevertheless, the proposed models have slightly better performance with respect to AUC and accuracy. It seems that more sophisticated models (e.g., SVMs and neural networks) do not necessarily produce more accurate predictions [7, 20].

**Table 2:** Model comparison for ATP single matches (moving window).

Model	AUC	Accuracy
Baseline	--	0.724
Logistic	0.691	0.727
SVM	--	0.719
Neural Network	0.657	0.724
LightGBM	0.688	0.723
Glicko	0.715	0.734
Glicko (Courts)	0.731	0.738

For the women’s matches, the results are very similar (Table 3), but the overall performances of men’s single matches are slightly better than those of women’s single matches. The Glicko model also has better performance in prediction, but adding the court-related variables does not cause any obvious improvements. Interestingly, the differences between the baseline model and statistical or machine learning models are very small. It seems that the ATP/WTA ranking contains plenty of information for predicting the winners of single matches in Grand Slam tournaments, and it is competitive against more complex models including all available variables. Additionally, similar to the results in previous studies, the prediction performances of all quantitative models are very similar.

**Table 3:** Model comparison of WTA single matches (moving window).

Model	AUC	Accuracy
Baseline	–	0.701
Logistic	0.656	0.699
SVM	–	0.700
Neural Network	0.648	0.701
LightGBM	0.667	0.700
Glicko	0.695	0.712
Glicko (Courts)	0.691	0.709

The prediction performances for the non-moving window case are approximately the same, and we show the results for the ATP and WTA in Table 4. The proposed Glicko models still have a small edge over all other models, but the advantage is smaller in the non-moving window case. This result is fairly reasonable since the Glicko model follows a Bayesian approach and the parameters should be updated according to its time sequence. On the other hand, the baseline model produces approximately the same prediction performances as the statistical and machine learning models. This indicates that the ranking covers almost all essential information for predicting the match results, since players selected to play in Grand Slam tournaments are quite homogeneous (in terms of playing strength). A line of evidence of homogeneity in the strength of players is the winning probability of higher-ranked players being at most 80%, as shown in Fig 2, and it is not easy to find over-whelming matches, even for the cases of top-seeded players vs. lower-ranked players.

**Table 4:** Model accuracy of single matches (non-moving window).

Model	ATP		WTA	
	AUC	Accuracy	AUC	Accuracy
Baseline	–	0.717	–	0.704
Logistic	0.685	0.718	0.661	0.704
SVM	–	0.721	–	0.709
Neural Network	0.700	0.720	0.690	0.707
LightGBM	0.705	0.717	0.694	0.707
Glicko	0.704	0.723	0.696	0.713
Glicko (Courts)	0.728	0.732	0.697	0.714

## Conclusion and discussion

Predictive analytics has recently become very popular, and many people are looking to construct models to explain observed phenomena in the form of “Observations = Model + Error”, which is often expressed as *y* = *f* (*x*_1_, *x*_2_, …, *x*_*k*_) + *ε*. There are two keys to constructing the models: selecting appropriate variables *x*_1_, *x*_2_, …, and *x*_*k*_ and choosing the proper functional form *f*(). Some studies focus on variable definition (or data structurization), while others emphasize enhancing model performance, depending on the study goal and the data availability. In this study, our goal is to find models with the highest accuracy in predicting winners of tennis matches, with the help of EDA to uncover important variables. Our conjecture is that once all relevant data are included, the role of model construction will be limited. This is analogous to “complete information” in economics, and the advantage of any players (or models) is very slim if all players have the same information.

Our study confirms this conjecture, and the results show that the models have a smaller impact on the prediction when the variable ranking is included. Many past studies had similar findings, and the prediction accuracies of all models were approximately the same e.g. [7, 21]. It seems that the ranking carries almost all information and adding more variables does not significantly increase the prediction accuracy. Thus, it is not surprising that the improvement by the proposed Glicko models is not large, with a prediction accuracy approximately 1% higher than that of the other models (approximately 70%). Nevertheless, the proposed approach provides the possibility of defining new variables without separating the models, for example, by different court surfaces. Of course, empirical results are often data dependent, and our findings do not necessarily apply to other ATP/WTA tournaments, since we think that the players’ strength in Grand Slam matches is fairly homogeneous.

The ranking variable provides competitive accuracy in predicting the winners of single matches. If we can treat the ranking as a sufficient statistic for prediction, then we may want to know, for example, whether sufficient statistics exist and how we define sufficient statistics. According to the seeding rules of Grand Slam matches, it seems that the public thinks that the ranking is a sufficient statistic and that the points earned in tournaments for the past 52 weeks are how the ranking should be decided. The Glicko model suggests an alternative index for measuring players’ strength. It would be interesting to explore whether we can improve the ATP/WTA ranking by applying a Bayesian approach.

Combining experts’ opinions and quantitative analysis is another way to construct prediction models. Relying solely on historical data and machine learning models overlooks the value of domain knowledge, ignoring the merit of human wisdom. However, only a few studies have mentioned the possibility of combining the prediction results of human judgements and data analysis [20]. On the other hand, experts’ opinions are similar to unstructured data, and it is difficult to format our thoughts into numerical values, i.e., perform structurization. This structurization process is crucial and would require personal intelligence and awareness of the problem. Perhaps we can adapt the idea used in the Delphi method, a popular method for structuring the opinions of a group of experts. Once human judgements are quantified, the remaining question might be how to properly weight human opinions and data analysis.

## Supporting information

S1 Table Hyper parameter and variables for each statistical and machine learning model. (PDF)
