# Correction: A study of forecasting tennis matches via the Glicko model

**DOI:** 10.1371/journal.pone.0314926

**Published:** 2024-12-02

**Authors:** Jack C. Yue, Elizabeth P. Chou, Ming-Hui Hsieh, Li-Chen Hsiao

An additional affiliation is missing for the first author. Jack C. Yue is also affiliated with the Center for Fundamental Science, Kaohsiung Medical University, Kaohsiung 80378, Taiwan
