# Supplementary material for: A study of forecasting tennis matches via the Glicko model
Source: PLoS One. 2022 Apr 8;17(4):e0266838. doi: 10.1371/journal.pone.0266838 (PMC8992979; doi:10.1371/journal.pone.0266838)
Supplement: S1 Table — (PDF) [file pone.0266838.s001.pdf]

S1 Table. Hyper Parameter and Variables for each Statistical and Machine Learning Model

Model	Hyper Parameter	Variables
Baseline model		Ranking
Proposed		Glicko,<br>cumulative scoring,<br>ranking,<br>age,<br>professional years
Logistic Regression		cumulative scoring,<br>ranking,<br>age,<br>professional years
SVM	RBF kernel,<br>C=3,<br>$\gamma = \frac{1}{\text{number of variables} \times \text{total variation}}$	cumulative scoring,<br>ranking,<br>age,<br>professional years
Neural Network	1 hidden layer,<br>80 neurons.<br>Active Function: hidden layer-ReLU, output layer-Sigmoid	cumulative scoring,<br>ranking,<br>age,<br>professional years,<br>dominant hand
LightGBM	boosting type = GBDT,<br>max depth = 10,<br>number leaves = 10,<br>learning rate = 0.03,<br>29 estimators	cumulative scoring,<br>ranking,<br>age,<br>professional years,<br>dominant hands
